# Epigenetic Treatment of Urothelial Carcinoma Cells Sensitizes to Cisplatin Chemotherapy and PARP Inhibitor Treatment

**DOI:** 10.3390/cancers13061376

**Published:** 2021-03-18

**Authors:** Sophia Thy, Alexandra Hommel, Sarah Meneceur, Anna L. Bartkowiak, Wolfgang A. Schulz, Günter Niegisch, Michèle J. Hoffmann

**Affiliations:** Department of Urology, Medical Faculty, Heinrich-Heine-University Duesseldorf, Moorenstr. 5, 40225 Duesseldorf, Germany; sophia.thy@hhu.de (S.T.); alexandra.hommel@hhu.de (A.H.); meneceur@hhu.de (S.M.); anbar120@hhu.de (A.L.B.); wolfgang.schulz@hhu.de (W.A.S.); michele.hoffmann@hhu.de (M.J.H.)

**Keywords:** urothelial carcinoma of the bladder, epidrug, romidepsin, PLX51107, BRCAness, HR deficiency, cisplatin resistance, PARP inhibitor, talazoparib, episensitization

## Abstract

**Simple Summary:**

Muscle-invasive urothelial carcinoma of the bladder (UC) is treated with chemotherapies based on the DNA-damaging drug cisplatin, which only works temporarily due to the development of drug resistance. In this study, we show that it may be possible to overcome such resistances by treating the cancer cells with specific epigenetic drugs. We investigated the “epidrug” PLX51107 that inhibits the chromatin regulator BRD4 (Bromodomain Containing 4). PLX51107 inhibited cell growth, caused DNA damage, and blocked DNA repair response in UC cells. Concomitant application of PLX51107 with cisplatin or the drug talazoparib, interfering with DNA repair, caused cell death very efficiently. PLX51107 thus sensitizes UC cells to other drugs and may allow therapy with novel effective anti-tumor drugs like talazoparib that normally only work in a small proportion of patients with specific gene mutations. These results may help to improve current standard therapy and to develop new treatment options urgently required for UC patients.

**Abstract:**

Muscle-invasive urothelial carcinoma (UC) is treated with cisplatin-based chemotherapy, which is only moderately efficient, mostly due to development of resistance. New therapy approaches are therefore urgently needed. Epigenetic alterations due to frequent mutations in epigenetic regulators contribute to development of the disease and to treatment resistance, and provide targets for novel drug combination therapies. Here, we determined the cytotoxic impact of the second-generation bromodomain protein inhibitor (BETi) PLX51107 on UC cell lines (UCC) and normal HBLAK control cells. PLX51107 inhibited proliferation, induced apoptosis, and acted synergistically with the histone deacetylase inhibitor romidepsin. While PLX51107 caused significant DNA damage, DNA damage signaling and DNA repair were impeded, a state defined as BRCAness. Accordingly, the drug strongly synergized with cisplatin more efficiently than romidepsin, and with the PARP inhibitor talazoparib to inhibit proliferation and induce cell death in UCC. Thus, a BETi can be used to “episensitize” UC cells to cytotoxic chemotherapy and inhibitors of DNA repair by inducing BRCAness in non BRCA1/2 mutated cancers. In clinical applications, the synergy between PLX51107 and other drugs should permit significant dosage reductions to minimize effects on normal tissues.

## 1. Introduction

Urothelial carcinoma (UC) of the bladder belongs to the top ten most common cancers in the world, with 573,278 new cases in 2020 [1]. Chemotherapy based on combinations of cisplatin with other cytotoxic drugs, e.g., gemcitabine, has been the standard of care for muscle-invasive UC for more than 30 years, but is only moderately efficient, so that the majority of patients progress or develop treatment resistance [2,3]. In previous work on mechanisms of cisplatin resistance, we have observed that UC cells (UCC) evade treatment-induced cell stress by various mechanisms [4]. In particular, substantial cellular plasticity may contribute to the development of treatment resistance in UCC [5]. Thus, in order to identify combination therapies that tackle resistance mechanisms, the cellular plasticity of UC needs to be restrained.

Cellular plasticity is mediated by dynamic epigenetic modifications regulating the silencing or activation of key genes [6]. Intriguingly, UC has one of the highest prevalences of alterations in epigenetic enzymes [7]. Thus, we explore the impact of epigenetic inhibitors (epidrugs) on UC cells and how they could be applied to “episensitize” towards approved standard chemotherapies. In particular, we have investigated compounds inhibiting histone deacetylases (HDACi) to define the HDAC isoenzymes most suitable as targets in UC and to uncover suitable combination partners [8,9,10]. In other solid cancers, clinical studies have revealed promising anti-cancer effects through episensitation, mostly by pan-HDACi, towards standard chemotherapy compounds [11,12]. We found, however, that pan-HDACi are less efficacious in UC, and rather propose the specific targeting of class I HDACs (mainly HDAC 1/2) for the treatment of this cancer [13,14]. Previously, we reported that combining the class I HDACi romidepsin with JQ1, an inhibitor of “bromodomain and extraterminal” proteins (BET), induced caspase-dependent apoptosis highly efficiently in UCC, in contrast to HDACi treatment on its own [8]. 

JQ1 is the best characterized BET inhibitor (BETi) and blocks the binding of proteins with bromodomains, especially of the bromodomain containing protein BRD4 to acetylated histones. BET proteins like BRD4 are considered “epigenetic readers” that link histone acetylation to transcriptional activation. BRD4 functions as transcriptional activator by recruiting the positive transcription elongation factor complex (P-TEFb) and RNA polymerase II [15,16]. BRD4 is overexpressed in UC tissues [17], correlating with grade, progression towards metastatic disease, and poor overall survival [18]. Anti-tumor activity has been demonstrated for various first-generation BETi (i.e., JQ1, iBET762, OTX015) in different preclinical models of solid and hematologic malignancies. Mechanistically, they dissociate BET proteins from super enhancers which results in the repression of oncogenes like *c-MYC* and an attenuation of plasticity and stemness [19,20,21]. BETi have also been investigated in clinical trials for hematopoietic and solid cancers, albeit mainly as a mono-treatment [22]. However, first-generation BETi caused dose-limiting hematologic and gastrointestinal toxicities, limiting their therapeutic index [15,23].

The second-generation BETi PLX51107 (in the following abbreviated PLX) used in the present study was developed with an improved therapeutic index and pharmaceutical profile [23]. PLX demonstrated potent anti-cancer activity in preclinical models of chronic lymphocytic leukemia and has been shown to cause growth inhibition and gene expression changes in a few other cancers such as melanoma [24]. This is the first study on the effects of PLX single treatment in UC cells. We observed reduced cell growth and the induction of apoptosis through the altered expression of cell cycle and apoptosis regulators. Similar to romidepsin [13,25], the BETi induced DNA damage and, at the same time, impaired DNA damage signaling by the downregulation of associated checkpoint proteins. 

Previously, the BETi JQ1 was reported to impair the non-homologous end joining (NHEJ) repair pathway in non-small cell lung cancer [26] and to downregulate components of the homologous recombination (HR) repair pathway, resulting in epigenetically induced “BRCAness” in *BRCA1* wild-type triple negative breast cancer (TNBC) cells [27]. BRCAness describes a defect in homologous recombination repair analogous to that induced by loss of *BRCA1* or *BRCA2* due to genetic alterations. *BRCA*-deficient cells are dependent on error-prone DNA-repair pathways like NHEJ, resulting in increased genomic instability and consequent sensitivity to DNA damaging agents and poly-(ADP)-ribose polymerase inhibitors (PARPi) [28]. PARPi inhibitors block the catalytic activity of PARP proteins and trap them on DNA, thereby interfering with replication and causing cell death in fast growing cancer cells [29]. We therefore studied the expression of various DNA repair molecules in PLX-treated UC cells with the aim of identifying suitable combinations partners for treatment of UC. We observed the downregulation of HR components and of *PARP1/2 (Poly(ADP-Ribose) Polymerase),* while NHEJ-associated factors were rather upregulated. These changes should sensitize UC cells to treatment with cisplatin or PARPi. Indeed, we found simultaneous treatment with PLX and cisplatin to act synergistically in UCC, but not in normal control cells. Different sequential treatment protocols were not superior to the concomitant application. Combination of chemotherapy compounds cisplatin or gemcitabine with PLX revealed better synergy profiles than their combination with the HDACi romidepsin or the combination of HDACi with PLX. In addition, the combination of PLX with the PARPi talazoparib acted highly synergistically in UCC to inhibit cell growth and induce cell death. In conclusion, we propose the consideration the BETi PLX51107 for combination with standard of care therapies for the treatment of UC. Furthermore, episensitation with PLX may allow for expanding the applicability of PARPi to *BRCA* wild-type cancer patients.

## 2. Materials and Methods

### 2.1. Cell Culture

Urothelial carcinoma cell lines VM-CUB1 and UM-UC-3, provided by the DSMZ (Braunschweig, Germany) and Dr. B. Grossman (Houston, TX, USA), were verified for identity and checked for mycoplasm contamination. The spontaneously immortalized normal human urothelial cell line HBLAK [30] was kindly provided by CELLnTEC (Bern, Switzerland). Cells were cultured as described previously [31]. 

Epidrugs, purchased from Selleckchem (Houston, TX, USA), were applied to the cells for up to 72 h. Solvent control cells were treated with corresponding DMSO concentrations. Cis-diamminedichloroplatinum-II (cisplatin) was obtained from Teva (Ulm, Germany) and talazoparib was purchased from Cayman Chemical (Ann Arbor, MI, USA). We measured cell viability by MTT assay (3-(4,5-Dimethylthiazol-2-yl)-2,5-diphenyltetrazoliumbromid; Sigma-Aldrich, St. Louis, MO, USA). For clonogenic assays, 1000 cells per six wells were reseeded from cells that had been treated with DMSO for 72 h. The same volume of cell suspension was then reseeded from the compound-treated cells without cell counting. Diluted cells were further cultured for at least 10 days and then stained with Giemsa to determine the long-term effect of epigenetic inhibitors and their combination treatment on cell proliferation. 

Caspase 3 and 7 activity was measured using Caspase-Glo 3/7 assay according to the manufacturer (1 h incubation time) with trypsinized cells in triplicate (Promega, Madison, WI, USA). The samples used were the same as those that were also submitted to flow cytometry analysis for apoptosis. To normalize caspase activity to cell number, the same trypsinized samples were used in parallel for CellTiter-Glo assay according to the manufacturer (10 min incubation time, Promega). A Victor multilabel plate reader was used to quantify luminescence (Wallac/Perkin Elmer, Rodgau, Germany). 

Acidic β-galactosidase staining was performed to stain senescent cells as described earlier [9]. 

### 2.2. Flow Cytometry

Cell cycle and cell death analyses were performed 72 h after the treatment of cells with indicated doses as previously described [32]. Briefly, collected attached and floating cells were stained with a Nicoletti buffer (50 µg/µl propidium iodide (PI), 0.1% sodium citrate, and 0.1% Triton X-100) for cell cycle analysis. Numbers of dead cells were determined after incubation with annexin V-FITC (Immunotools, Friesoythe, Germany) in annexin V binding buffer and PI (2 μg/mL). Flow cytometry analysis was performed using a Miltenyi MACSQuant® analyzer (Milteny Biotec GmbH, Bergisch Gladbach, Germany) and MACSQuantify software.

### 2.3. Protein Expression Analysis

UCC were treated for 72 h with cell line-specific half maximal inhibitory concentration (IC_50_) doses of PLX before cell lysates were prepared. Immunoblot analysis was performed with whole cell extracts as described in [8]. Antibodies for target detection and secondary antibodies are listed in Table S1. Targets were visualized by SuperSignal™ West Femto (Thermo Scientific, Rockford, IL, USA) and WesternBright Quantum kit (Biozym, Hessisch Oldendorf, Germany).

Immunocytochemistry was performed as described in detail elsewhere [32]. Briefly, cells were treated on coverslips with the indicated doses, fixed with formaldehyde, permeabilized, and incubated in blocking solution. Cells were stained by the application of 50 μL of primary antibody solution per coverslip (Table S1) and incubated at 4 °C overnight. After washing, secondary antibodies were likewise added at room temperature for 1 h. After washing, the nuclei were counterstained with DAPI (4′,6-Diamidin-2-phenylindol). Finally, mounting medium (Dako, Santa Clara, CA, USA) was added. Images were taken by means of an Olympus Fluoview FV-1000 microscope (Hamburg, Germany), kindly provided by the Center for Advanced Imaging, Heinrich Heine University (Cai, numerical aperture 1/20, 60× objective). Images were processed using the Fluoview FV-1000 software, version 3.1. 

### 2.4. RNA Expression Analysis

UCC were treated for 72 h with cell line-specific IC_50_ doses of PLX before cell lysates were harvested for RNA extraction. Extraction was performed using the RNeasy mini kit according to the manufacturer (Qiagen, Hilden, Germany). cDNA was generated from one μg of RNA by means of the FastGene Scriptase II cDNA kit (NIPPON Genetics Europe, Düren, Germany). Quantitative reverse transcription polymerase chain reaction (qRT-PCR) was performed with either Luna^®^ Universal qPCR Master Mix (New England Biolabs, Frankfurt, Germany) using a two-step temperature profile according to the manufacturer’s instructions, or with QuantiTect SYBR Green RT-PCR Kit (Qiagen, Hilden, Germany) using a three-step program on the LightCycler 96^®^ platform (Roche, Grenzach-Wyhlen, Germany) or the BioRad CFX384 platform (Bio-Rad, Hercules, CA, USA). The housekeeping gene TATA-box binding protein (*TBP*) was measured as a reference. Primer sequences are given in Table S2. 

### 2.5. Calculation of IC_50_ Values, Drug Synergy and Statistics

Dose response curve analysis was performed by means of an MTT assay after 72 h. IC_50_ concentrations were calculated by means of the AAT-Bioquest^®^ online calculator tool (https://www.aatbio.com/resources/ (accessed on 17 March 2021)). IC_50_ values for all compounds used in this study are given in Table S3. 

To determine drug synergy for the combined treatments, compounds were applied to the cells in fixed dose ratios. DMSO treated cells served as solvent controls. Based on the IC_50_ values of the individual drugs for each cell line, individual dose ratios were chosen for fixed ratios. Thus, cells were treated with 0.125-fold, 0.25-fold, 0.5-fold, 0.75-fold, 1-fold, 1.5-fold, and 2-fold amounts of the cell line-specific IC_50_ dosage for compound A alone, likewise for compound B alone. For the combined treatment, the cells were treated, e.g., with 0.5-fold IC_50_ of compound A, plus 0.5-fold IC_50_ of compound B. Seven different combinations of concentrations were applied and then analyzed by the Chou–Talalay method using the CompuSyn software [33]. Based on the combination index (CI) equation, synergism (CI < 1), additive effect (CI = 1), or antagonism (CI > 1) were determined by analyzing a series of doses and the according effect for a drug alone and for drug combinations. Using this dose/effect correlation, a plot is simulated by the software. To achieve a better resolution for the plots, they were redrawn by GraphPad based on the results given in the Compusyn report generated by the software. 

GraphPad Prism version 8 was used for data calculation and the generation of graphs. 

## 3. Results

Since BRD4 is one of the main targets of BETi, we determined its gene expression across 17 UCC and 2 different cultures of normal urothelial control cells (HBLAK and TERT-NHUC, Figure S1a). *BRD4* gene expression was detectable in all 17 UCC to a variable extent and also in normal control cells. We have reported detectable protein expression across 10 UCC and the two normal control cell lines earlier [8]. We chose two UCC, namely UM-UC-3 and VM-CUB1, for analyses on the impact of the second-generation BETi PLX51107, alone and in combination with the class I-specific HDACi romidepsin, as well as for further putative combination treatment approaches in this study. The two UCC displayed robust expression of BRD4 and have been extensively analyzed in various previous studies on the impact of HDAC inhibitors [25,34,35] and the first-generation BETi JQ1 [8]. Notably, VM-CUB1 and UM-UC-3 cells responded significantly differently to JQ1 with a 10-fold higher IC_50_ dose for UM-UC-3 cells. Thus, these two cell lines represent the sensitive and resistant groups of UCC, respectively. The likewise well-characterized HBLAK cell line, originating from a spontaneously immortalized primary culture of normal uroepithelial cells [30], was used as a normal control.

### 3.1. BET Inhibitor PLX51107 Inhibits Proliferation of UC Cells and Disturbs Cell Cycle Regulation

Initially, dose response curves were established for the effect of PLX on the UC cells. The IC_50_ dose of PLX was fourfold higher for UM-UC-3 cells (8.8 µM) than for VM-CUB1 (2 µM). In contrast to JQ1, HBLAK cells were more sensitive to PLX than the UCC with an IC_50_ dose of 0.6 µM (Table S3). To check for the molecular effects of PLX IC_50_ doses, we analyzed the expression of established BETi target genes. While *c-MYC* (MYC proto-oncogene) has been reported as a main target of BETi [19], its expression is not altered by treatment of UC cells with JQ1 [8]. Concurringly, c-MYC expression was not significantly altered by PLX in UCC (Figure S1b). In contrast, *HEXIM1 (HEXIM P-TEFb Complex Subunit 1),* another typical target gene of BETi [36], was strongly induced in the two investigated UCC by PLX (Figure S1c) and in HBLAK control cells. In conclusion, IC_50_ doses of PLX resulted in the expected gene expression changes in UCC. 

**Figure 1:**
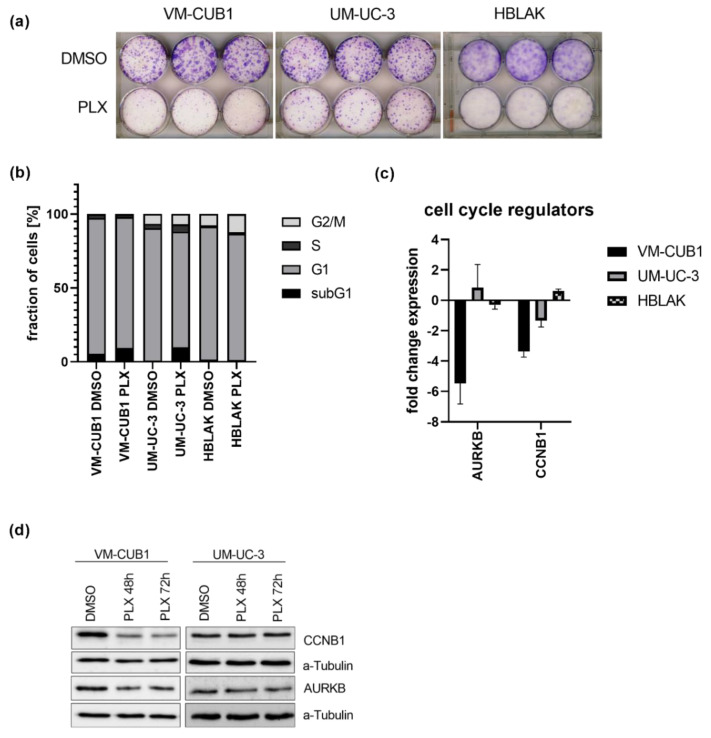
Impact of PLX51107 treatment on proliferation and cell cycle. (**a**) Long-term effects on cell proliferation were investigated by clonogenicity assays. Cells were treated for 72 h with cell line-specific IC_50_ doses (VM-CUB1 2 µM, UM-UC-3 8.8 µM, HBLAK 0.6 µM) or DMSO, subsequently highly diluted, and further cultured for 10 days. Cell colonies were stained with Giemsa. (**b**) Cell cycle alterations were monitored by PI (propidium iodide) staining and flow cytometry 72 h after treatment with IC_50_ doses of PLX. RNA expression 72 h after treatment with IC_50_ doses (**c**) and protein expression 48 h and 72 h after treatment (**d**) of cell cycle regulators CCNB1 and AURKB were determined by qRT-PCR and western blot analysis, respectively. α-Tubulin served as a loading control for western blot analysis. RNA expression of indicated target genes was normalized to the reference gene *TBP (TATA-Box Binding Protein)*. Expression changes are displayed as fold change relative to the respective DMSO control.

The long-term effect of the cell line-specific IC_50_ doses of PLX on cell proliferation were determined by clonogenicity assays. Proliferation was more strongly inhibited in VM-CUB1 and HBLAK cells compared to UM-UC-3 (Figure 1a and Figure S2a). PLX-treated HBLAK cells appeared to develop a senescent morphology and stained positive for acidic β-galactosidase as a marker for senescence (Figure S2b). Cell cycle analysis by flow cytometry after PLX treatment demonstrated an increase of mainly subG1 cells at the expense of the G1 phase. In contrast to HBLAK, which slightly accumulated in the G2/M phase, no severe cell cycle alterations were detectable in UCC after three days (Figure 1b) and six days (Figure S3). As representatives of G2/M activity, we further measured expression of cyclin B1 (*CCNB1*) and aurora kinase B (*AURKB*) after treatment with PLX (Figure 1c). Both genes were significantly downregulated in VM-CUB1 but neither in UM-UC-3 cells nor in HBLAK control cells (Figure 1c). The same results were obtained by protein analysis (Figure 1d, Figure S2c). We noted already earlier that UM-UC-3 cells respond to other epidrugs, e.g., HDAC inhibitors with a different kinetic compared to VM-CUB1 cells. This applied also to the extent of AURKB downregulation after treatment with different class I-specific HDACi. However, our very recent analysis of mutations in cell cycle and checkpoint control genes did not reveal differences between the two cell lines with the exception of *CDKN2A* (*Cyclin Dependent Kinase Inhibitor 2A*) that is homozygously deleted in UM-UC-3, but mutated in VM-CUB1 [13]. Taken together, even though PLX treatment induced only mild cell cycle alterations compared to romidepsin [13,25], we detected deregulation of cell cycle genes in VM-CUB1 cells, which are more sensitive to BETi than the resistant UM-UC-3 cells.

### 3.2. PLX51107 Treatment Results in Induction of Apoptosis and Interferes with Regulators of Cell Death

**Figure 2:**
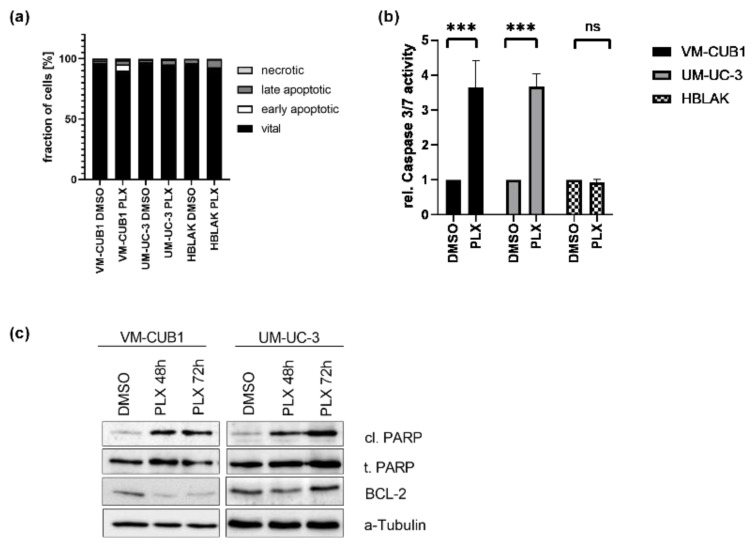
Induction of cell death in UCC after treatment with BETi PLX51107. (**a**) Induction of cell death was measured by annexin V/PI staining and flow cytometry 72 h after treatment with cell line-specific IC_50_ doses of PLX or DMSO. Percentages of viable, early apoptotic, late apoptotic, and necrotic cells are displayed in bar graphs in different shades of gray as indicated. (**b**) Likewise, the activity of caspase 3 and 7 was measured in the same samples used for flow cytometry by luminometric caspase assay. In parallel, the samples were used for a luminometric proliferation assay to normalize caspase activity to cell numbers (rel. caspase activity). Further normalization to respective DMSO controls (set as 1) was applied. *** denotes *p* < 0.001. ns denotes not significant differeneces. (**c**) PARP cleavage (cl. PARP) was determined by western blot analysis after treatment for 48 h and 72 h with PLX. Total PARP protein (t. PARP) was detected as a loading control. Anti-apoptotic protein BCL2 was detected, while α-Tubulin served as a loading control.

As expected from the increased numbers of subG1 cells following PLX treatment, cell death analysis by annexin V/PI-staining and flow cytometry demonstrated that VM-CUB1 cells responded more strongly to PLX by apoptosis induction than UM-UC-3 cells (Figure 2a). HBLAK cells were also less strongly affected. Concurringly, the activity of caspase 3 and 7 was strongly increased by PLX in both UCC, but not in HBLAK cells (Figure 2b). Since caspase activity may result in PARP cleavage, we performed western blot analysis. Expectedly, PARP cleavage increased in both UCC over time (Figure 2c), but not in HBLAK cells (Figure S2b). Furthermore, BCL2 (BCL2 Apoptosis Regulator), which is known to promote apoptosis resistance of UC cells [37], became downregulated, particularly in VM-CUB1 cells. 

### 3.3. BET Inhibition Causes DNA Damage, but Not Activation of DNA Damage Signaling

**Figure 3:**
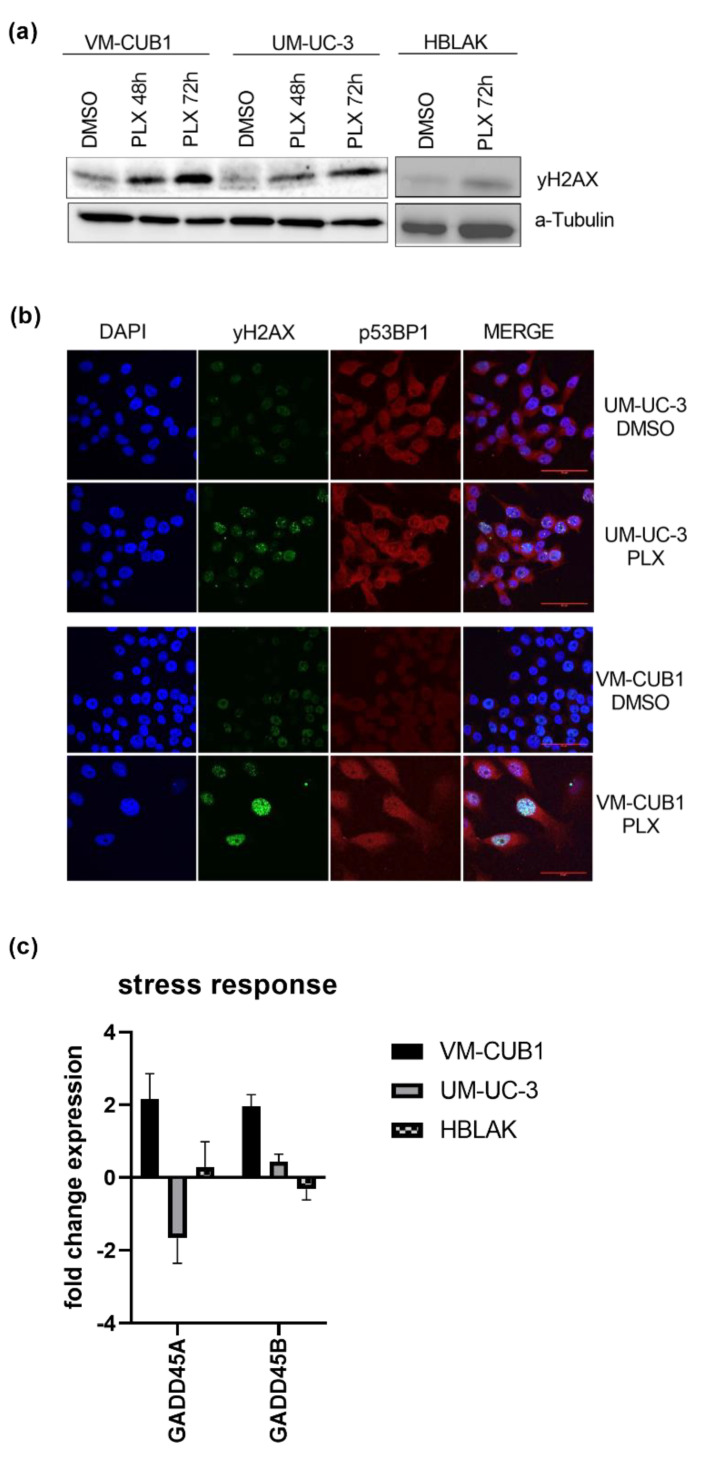
Induction of DNA damage by BETi treatment. (**a**) Increased phosphorylation of γH2AX as a marker of DSB (double strand breaks) was determined by western blot analysis after treatment for 48 h and 72 h with cell line-dependent IC_50_ doses of PLX. A-Tubulin served as a loading control. (**b**) Phosphorylated γH2AX (green fluorescence) and p53BP1 (red fluorescence) were stained by immunocytochemistry and imaged by means of confocal microscopy (scale bar = 50 µm). Background from secondary antibodies is shown in Figure S1d. (**c**) RNA expression 72 h after treatment with IC_50_ doses of PLX was measured for the stress response markers *GADD45A* and *GADD45B* by qRT-PCR. RNA expression of indicated target genes was normalized to the reference gene *TBP*. Expression changes are displayed as fold change relative to the respective DMSO control.

Since impaired DNA repair has been reported after BET inhibition [26,27], we investigated the effects of PLX on DNA damage signaling in UCC in this study. Phosphorylated γH2AX (H2A.X Variant Histone) as a marker of DNA double strand breaks (DSB) was significantly increased after PLX treatment of both UCC, but less strongly in HBLAK cells as indicated by western blot analysis (Figure 3a). These results were confirmed by immunocytochemistry using the double staining of γH2AX and p53BP1 (P53-Binding Protein 1) as a second marker for DSB. Both cell lines displayed accumulation of γH2AX foci following PLX treatment which was particularly strong in VM-CUB1 cells (Figure 3b). In addition, we found significant induction of *GADD45A* and *GADD45B* (*Growth Arrest And DNA Damage Inducible Alpha/Beta*) gene expression in treated VM-CUB1 cells, but not in UM-UC-3 and HBLAK cells (Figure 3c). Both genes can be induced in response to cell cycle stress and after treatment with DNA-damaging agents [38].

**Figure 4:**
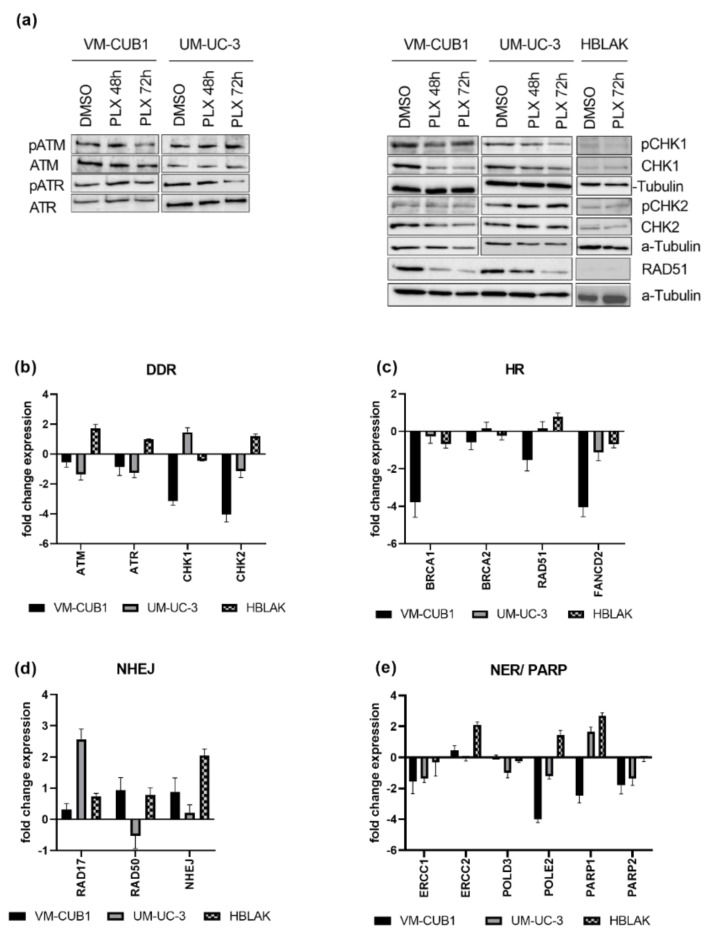
PLX impairs DNA damage signaling and induces DNA repair deficiency. (**a**) Activation of checkpoint proteins through phosphorylation was determined by western blot analysis after treatment for 48 h and 72 h with cell line-specific IC_50_ doses of PLX. Respective total proteins and α-Tubulin were detected as loading controls. Likewise, RNA expression was determined in (**b**) by qRT-PCR 72 h after PLX treatment. RNA expression of key components of HR (homologous recombination) (**c**) was measured accordingly. RNA expression of NHEJ (non-homologous end joining) (**d**), NER (nucleotide excision repair), and *PARP* genes (**e**) was measured by qRT-PCR and normalized to the reference gene *TBP*. Expression changes are displayed as fold change relative to the respective DMSO control.

Next, we analyzed the activation of DNA damage signaling following PLX treatment. Intriguingly, despite the induced DNA damage, we could detect neither the activation of the ATM (ATM Serine/Threonine Kinase) or ATR (ATR Serine/Threonine Kinase) DNA damage signaling kinases, nor the checkpoint kinases CHK1 (Checkpoint Kinase 1) and CHK2 (Checkpoint Kinase 2) in the UCC. In fact, in VM-CUB1 cells, total protein expression of CHK1 and CHK2 was even downregulated (Figure 4a). Neither changes in total protein nor phosphorylation of checkpoint kinases was detectable in PLX-treated HBLAK cells. Concurringly, *CHK1* and *CHK2* RNA expression was also strongly downregulated in VM-CUB1 cells (Figure 4b). In comparison, UM-UC-3 cells rather downregulated *ATM* and *ATR* expression. In contrast, HBLAK cells responded to PLX by the slight induction of *ATM, ATR,* and *CHK2* RNA expression.

### 3.4. DNA Damage Repair Is Impaired in UC Cells Treated with PLX51107 

Since, in addition to DNA damage signaling, DNA repair might also be impaired by BET inhibition, we measured gene expression changes of DNA damage repair components after PLX treatment (Figure 4c–e). We investigated DNA damage repair pathways that are generally involved in DSB repair, e.g., homologous recombination (HR) and non-homologous end joining (NHEJ), and pathways that are particularly involved in the repair of cisplatin-induced damage, e.g., HR and nucleotide excision repair (NER). Key components of HR such as *BRCA1 (BRCA1 DNA Repair Associated)*, *RAD51 (RAD51 Recombinase),* and *FANCD2* (*FA Complementation Group D2*) were downregulated by PLX, particularly in VM-CUB1 cells. *BRCA2* (*BRCA2 DNA Repair Associated*) remained rather unchanged (Figure 4c). Expression changes in normal HBLAK cells were limited. RAD51 downregulation in UCC was also confirmed on the protein level, while it was hardly detectable in HBLAK (Figure 4a). Genes encoding components of the NHEJ machinery (*RAD17, RAD50, NHEJ; RAD17 Checkpoint Clamp Loader Component*; *RAD50 Double Strand Break Repair Protein*, *Non-Homologous End-Joining Factor 1*) were mainly induced in UCC (Figure 4d) and HBLAK. NER factor *ERCC1* (ERCC Excision Repair) was downregulated in both UCC, while *ERCC2* was not significantly altered. In contrast, *ERCC1* was only slightly changed in HBLAK control cells, while *ERCC2* was strongly induced (Figure 4e). *POLD3 (DNA Polymerase Delta 3)* was downregulated in UM-UC-3, *POLE2 (DNA Polymerase Epsilon 2)* was strongly decreased in both UCC (Figure 4e), but significantly induced in HBLAK. Taken together, PLX induced HR deficiency and rather activated NHEJ in UCC. 

Furthermore, genes that function in the repair of cisplatin induced DNA damage, and which may contribute to cisplatin resistance when overexpressed, became downregulated by PLX treatment in UCC. Poly(ADP-ribose) polymerases (PARP1/2) are activated by DNA breaks and are needed for further repair signaling [29]. *PARP1* was reduced in VM-CUB1 cells but induced in UM-UC-3 and HBLAK. *PARP2* was decreased by PLX in both UCC and remained unchanged in HBLAK (Figure 4e). In summary, PLX altered the expression of a variety of DNA repair factors, which likely contributes to DNA repair deficiency. Importantly, HBLAK control cells responded less strongly or displayed expression changes in the opposite directions, raising the possibility that UCC may be more vulnerable to further DNA damaging treatment than normal cells. HR-deficient cells have been reported to be sensitive to PARP inhibitors [39,40,41], and synthetic lethality is dependent on active NHEJ [42]. Obviously, PLX treatment altered expression of the DNA damage machinery in UCC towards a state that may render UCC sensitive to PARP inhibition. In addition, downregulation of PARP enzyme expression may make UCC super-dependent on the residual PARP protein so that they might become sensitized to PARP inhibitors by treatment with PLX. 

With regard to cisplatin-induced damage repair in particular, we also found some components of the NER pathway downregulated by PLX. Downregulation of *FANCD2,* as described above, may further impair the repair of cisplatin-induced crosslinks [43]. Thus, PLX treatment might also impair the repair of cisplatin-induced DNA damage and force UCC to undergo cell death. In conclusion, we hypothesized that combined treatment of PLX with PARP inhibitors or cisplatin might induce synthetic lethality in UC cells. 

### 3.5. Combination Treatments with Epigenetic Inhibitors Have Synergistic Effects in UC Cells

In previous work we found that combined treatment with the first-generation BETi JQ and the class I-specific HDACi romidepsin acted synergistically in various UCC but spared normal HBLAK cells [8]. We had also reported earlier on the response of UC cells towards romidepsin alone [9,13,25]. We therefore investigated the combination of PLX and romidepsin for synergy, using the same concentrations of the HDACi as in our previous work (Figure S4a). Indeed, analysis according to the Chou–Talalay method revealed dose-dependent synergy for the new combination in both UCC [33]. The Chou–Talalay method was used to define the window of optimal drug dosages (1) with sufficient efficacy, (2) allowing application of minimal dosages and thereby (3) differentiating between cancerous cells and benign controls in order to avoid toxicity. As expected, the more PLX-sensitive VM-CUB1 cell line responded more strongly than UM-UC-3. Doses of 1 µM PLX and 2.15 µM romidepsin and higher acted synergistically on VM-CUB1 cells, combination dosages ≥ 4.4 µM PLX and 1.7 µM romidepsin and higher acted synergistically on UM-UC-3 cells. For both cell lines, four of the seven analyzed combination doses were strongly synergistic (CI ≤ 0.17 VMCUB1, CI ≤ 0.60 UM-UC-3). Increased effect rates corresponded with increased dosage. Unfortunately, unlike with the old drug combination of romidepsin and JQ1, treatment of normal HBLAK control cells with HBLAK-specific IC_50_ doses acted also synergistically at lower doses (lower value of fraction affected) and antagonistically at higher doses (higher value of fraction affected). Antagonistic effects started from doses of 0.6 µM PLX and 0.9 µM romidepsin. Thus, even though synergistic action in UCC might allow dose reduction for the combined treatment with PLX and romidepsin, this may affect normal cells more than the combination of JQ1 with romidepsin.

Next, we followed up on our hypothesis that PLX might sensitize UCC to cisplatin. We therefore investigated the treatment responses to the combination of PLX with cisplatin, comparing it with the combination of cisplatin with the HDACi romidepsin. Combined simultaneous treatment with romidepsin and cisplatin had only weak synergistic effects in VM-CUB1 that were highly dose-dependent and were only achieved at doses equal to or above 0.75-fold of the IC_50_ dosages (Figure S4b). Synergism was also achieved for HBLAK cells with a dosage around the IC_50_, resulting in 80 % of dead cells, indicating high toxicity of the treatment to normal cells. In UM-UC-3, effects were only additive or antagonistic. Generally, sequential rather than concomitant treatment with romidepsin and cisplatin did not significantly enhance synergism. Pretreatment for 24 h with romidepsin improved synergism in UM-UC-3 cells, but also in HBLAK (Figure S4c). Treatment with the HDACi 24 h after cisplatin application killed HBLAK cells most strongly (Figure S4d). 

**Figure 5:**
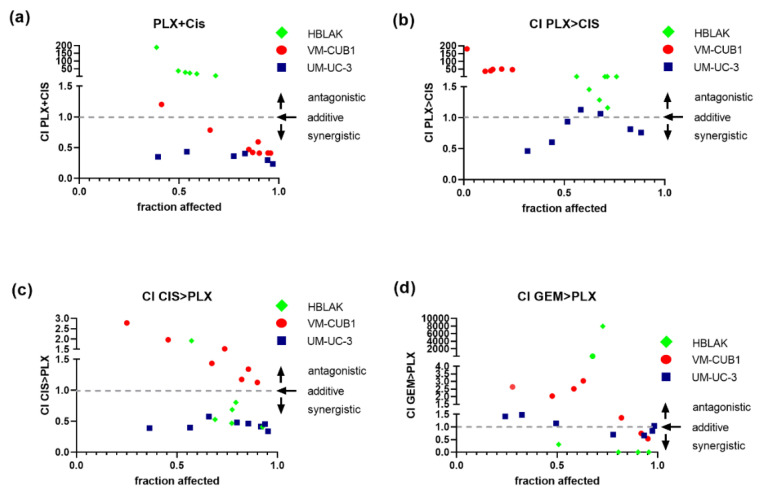
Synergistic effects of combination treatment with PLX and chemotherapeutic compounds on UCC. Cell viability was measured at constant dose ratios by MTT assay 72 h after the indicated simultaneous or sequential treatment (cell line-specific treatment dosages are given Table S4). Combination index (CI) plots for the combination of PLX and cisplatin (CIS) or gemcitabine (GEM) were calculated according to the Chou–Talalay method with Compusyn software. Combination index versus the number of dead cells (fraction affected) is displayed. CI < 1 indicates synergism (dashed line), CI = 1 additive effects and CI > 1 antagonism. (**a**) CI for simultaneous treatment with PLX and CIS, (**b**) CI for 24 h of pre-treatment with PLX followed by application of CIS for a further 48 h, (**c**) CI for 24 h of pre-treatment with CIS followed by the application of PLX for a further 48 h, (**d**) CI for sequential treatment with PLX and GEM are plotted. Green rhombi denote HBLAK, red circles VM-CUB1, and blue squares UM-UC-3 data points, respectively.

In comparison, simultaneous treatment with PLX and cisplatin performed much better and was strongly synergistic, particularly in the less PLX-sensitive UM-UC-3 cell line, were all seven dose combinations achieved intriguingly low CI values (Figure 5a, VM-CUB1 CI range 0.41–0.78, UM-UC-3 CI range 0.43–0.23). In UM-UC-3 cells, a reduced dosage of 0.125-fold IC_50_ values (1.1 µM PLX and 0.5 µM cisplatin) already resulted in strong synergism (CI = 0.35). In VM-CUB1 cells, 0.25-fold IC_50_ doses (0.5 µM PLX and 1.25 µM cisplatin) and higher acted synergistically. No synergism was observed in HBLAK cells, even at 2-fold IC_50_ dosages, which may be advantageous during in vivo application. As with romidepsin, sequential treatment was not superior, sometimes even abolishing synergism or killing HBLAK control cells more strongly (Figure 5b,c). Sequential combination treatment with gemcitabine and PLX (Figure 5d) was again not superior to simultaneous treatment with cisplatin and PLX (Figure 5a). 

**Figure 6:**
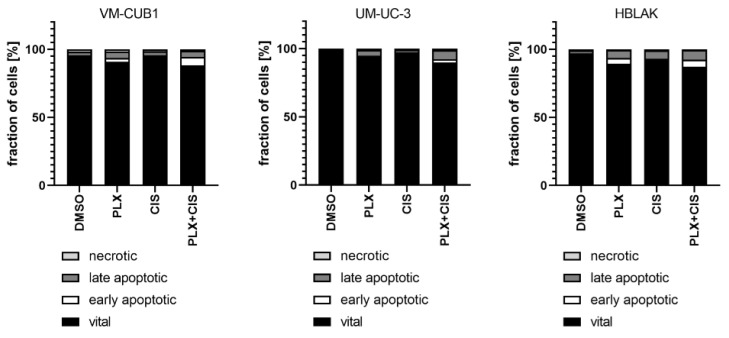
Induction of cell death after combination treatment with PLX and cisplatin at a reduced dosage. Induction of cell death was measured by annexin V/PI staining and flow cytometry 72 h after treatment with a reduced dosage. All cell lines were treated with 0.5-fold IC_50_ concentrations of sensitive VM-CUB1 cells (1 µM PLX, 2.5 µM CIS). Percentages of viable, early apoptotic, late apoptotic, and necrotic cells are displayed in bar graphs in different shades of gray as indicated.

According to the results of the synergism analysis, we investigated how UCC and normal HBLAK cells would be affected by combination therapy with a reduced dosage of PLX and cisplatin. Thus, we applied the 0.5-fold IC_50_ doses of VM-CUB1 (1 µM PLX and 2.5 µM cisplatin) to all cell lines. Flow cytometry analysis demonstrated increased induction of cell death by combined low dose treatment compared to single treatment in UCC, which was not further increased by the combination in HBLAK cells (Figure 6). Reduced dosage also still effectively inhibited long-term growth of UCC over 10 to 14 days (Figure S5a). Thus, we identified a new highly effective treatment approach for UCC with reduced normal toxicity by combining the BETi PLX51107 with the chemotherapy compound cisplatin. 

**Figure 7:**
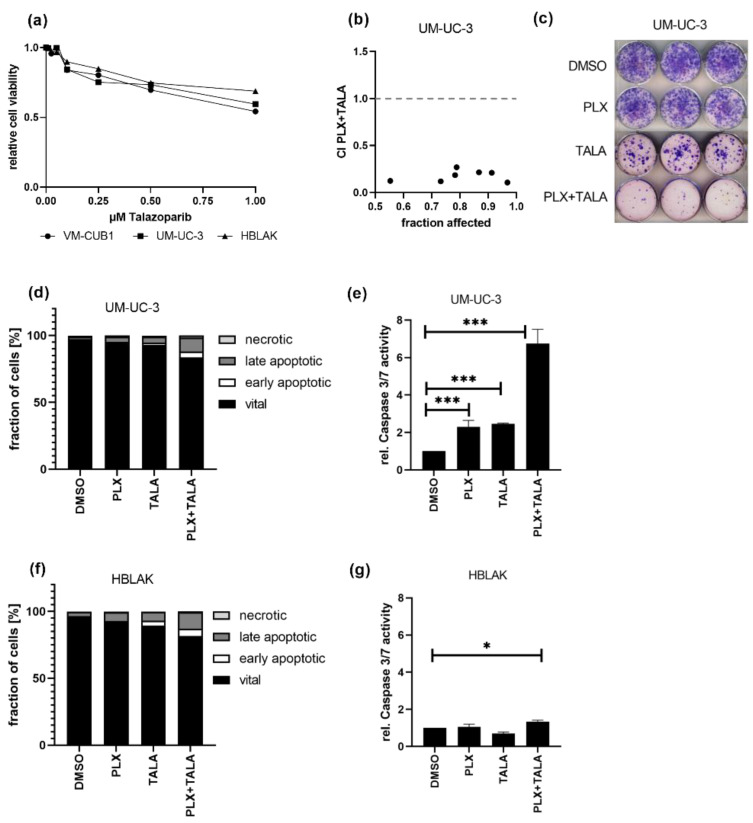
Strong synergistic action of combined treatment with PLX and PARPi talazoparib. (**a**) Cell viability 72 h after mono-treatment with indicated doses of talazoparib was measured by MTT assay. Relative cell viability normalized to respective DMSO controls is displayed. (**b**) CI plot for simultaneous treatment of UM-UC-3 with PLX and talazoparib (TALA) for 72 h. Applied combination dosages are given in Table S4. (**c**) Clonogenic growth following treatment with reduced drug concentrations (0.6 µM PLX, 0.5 µM TALA). (**d**) Induction of cell death was measured by annexin V/PI staining and flow cytometry 72 h after treatment with a reduced dosage. Percentage of viable, early apoptotic, late apoptotic, and necrotic cells is displayed in bar graphs in different shades of gray as indicated. (**e**) Activity of caspase 3 and 7 was measured in the same samples used for flow cytometry by luminometric caspase assay. In parallel, the samples were used for a luminometric ATP assay to normalize caspase activity to cell numbers (rel. caspase activity). Further normalization to respective DMSO controls (set as 1) was applied. *** denotes *p* < 0.001, * *p* < 0.05. Likewise, cell death induction was analyzed for HBLAK cells (**f**,**g**).

Finally, we investigated whether the combination of the BETi PLX with a PARP inhibitor might also be promising as a treatment approach for UC cells. Dose response curves for the PARPi talazoparib (TALA) reflected that both UCC and HBLAK tolerate the PARPi alone quite well, sharing an IC_50_ value of 1 µM (Figure 7a). Thus, we decided to further analyze the weakly PLX-sensitive UM-UC-3 cell line for response to this combination. Intriguingly, this combination treatment killed UM-UC-3 cells very efficiently in a highly synergistic manner even at the lowest applied dose combination of 0.125-fold IC_50_ (1.1 µM PLX and 0.125 µM TALA) (Figure 7b, CI range 0.10–0.26). These results were further confirmed by experiments in which we applied an even more reduced dosage using the HBLAK-specific 0.5-fold IC_50_ dose of talazoparib (0.5 µM) and the 1-fold IC_50_ value for PLX (0.6 µM). This combined treatment strongly inhibited long-term cell growth compared to mono-treatment (Figure 7c). Furthermore, it resulted in significantly more dead UM-UC-3 cells than the single treatments, even though we applied only 0.07-fold of the IC_50_ of PLX to usually rather PLX-insensitive UM-UC-3 (IC_50_ PLX 8.8 µM, Figure 7d). Concurringly, caspase activation was strongly increased in UM-UC-3 cells (Figure 7e) but not in HBLAK (Figure 7f,g), suggesting that the combination treatment may result in tolerable normal toxicity. With this combination, even sequential treatment remained synergistic when a reduced dosage was applied to PLX-insensitive UM-UC-3 cells (Figure S5b). Taken together, we discovered another highly effective drug combination where drug synergism allowed for significantly decreased doses for the treatment of UCC.

## 4. Discussion

New therapy approaches are urgently needed to improve the outcome of UC patients. One promising approach employs combinations with epigenetic inhibitors that sensitize towards established chemotherapeutic drugs by limiting the plasticity and treatment evasion of cancer cells. Promising anti-cancer effects through “episensitation” of this kind have already been demonstrated in clinical studies for several solid cancers, mostly by using pan-HDACi with standard chemotherapy compounds [11,12]. 

In UC, the effect of pan-HDACi and even of compounds like romidepsin that specifically inhibit the proliferation-associated class-I HDACs is however limited. BETi such as JQ1 synergize with HDACi to more efficiently and specifically kill UC cells [8]. In this study, we therefore investigated whether the second-generation BETi PLX51107 would synergize with romidepsin and, more broadly, episensitize UC cells to cisplatin, the most widely employed chemotherapy drug in UC. Both combinations indeed proved synergistic. Moreover, analysis of gene expression changes induced by PLX suggested that it would synergize with inhibitors of DNA repair, which we also confirmed for the PARP inhibitor talazoparib. Our investigation therefore identifies several promising new drug combinations for further development.

The second-generation BETi PLX51107 is less well investigated than JQ1. PLX has been investigated in 11 publications on a few cancer entities (PubMed query on 23rd January 2021), reporting on growth inhibition and gene expression changes [24]. Currently two clinical studies are registered at www.clinicaltrials.gov, accessed on 1 May 2020. One study applied PLX as a mono-treatment for advanced malignancies (NCT02683395). The other investigates the combination of PLX with the DNA methyltransferase inhibitor azacytidine (NCT04022785). Thus, while the drug is clearly promising, further research is needed, especially in UC. This prompted us to characterize the effects of PLX single treatment on UC cells in this study to determine whether it is superior to JQ1 and possibly suitable for combination therapies with other epidrugs or for episensitization. As for JQ1, we found that VM-CUB1 cells were highly sensitive at a low IC_50_ concentration compared to the rather resistant UM-UC-3. In contrast to JQ1, the IC_50_ dose for PLX was lower in control HBLAK cells than in UCC. Thus, PLX alone, like HDACi alone [25], may also be toxic for normal cells. This may also explain, why the combined treatment with PLX and romidepsin performed worse than the combination of JQ1 and romidepsin, which spared normal uroepithelial cells better. This limitation might be overcome by highly synergistic combination treatment approaches that allow significant dose reduction while still efficiently killing cancer cells. Combinations of HDACi and JQ1 also do not induce apoptosis in other normal cells, including melanocytes, embryonic fibroblasts, and hematopoietic progenitor cells [44,45,46]. For PLX, no investigations on the response of other normal cells have been published, to our knowledge. 

As observed for JQ1 in UCC, *c-MYC* expression was not affected by PLX. A second BETi response marker, *HEXIM1* [36], responded strongly to PLX, both in UCC and HBLAK. Long-term proliferation was reduced in both UCC and HBLAK cells. In UCC, cell cycle analysis demonstrated an increase of subG1 cells rather than significant cell cycle alterations, whereas PLX-treated HBLAK cells accumulated to some extent in G2/M. Concurringly, cell cycle regulators like *CCNB1* and *AURKB* responded differently in UCC compared to normal HBLAK. Mono-treatment with PLX induced apoptosis in VM-CUB1 more strongly than in UM-UC-3, but also some cell death in HBLAK. However, we could detect neither increased caspase activity in PLX treated HBLAK cells nor PARP cleavage. Instead, PLX treated HBLAK cells stained positive for a senescence marker suggesting that the PLX response of HBLAK cells is dominated by reduced proliferation and senescence rather than apoptosis induction, which would limit damage to normal cells in tissues. Treatment of other solid cancer types with PLX also resulted in reduced cell proliferation. Primary cultures from anaplastic thyroid cancers were even more sensitive to PLX with IC_50_ doses around 1 µM. Mono-treatment with this BETi reduced cell growth and c-MYC expression. Apoptosis induction, however, appeared to be cell line dependent. Tumor growth was also reduced in vivo [47]. In renal carcinoma cell lines, PLX decreased cell viability only at relatively high IC_50_ concentrations, between 7–10 µM, and also decreased c-MYC expression dose-dependently [48]. Tumor growth in a xenograft model was also decreased. Likewise, analysis in a V600E melanoma syngenic model demonstrated delayed melanoma tumor growth in vivo [49]. Tiago et al. compared the impact of JQ1 (1 µM) and PLX (2 µM) on *BRAF (B-Raf Proto-Oncogene*) -mutant melanoma cell lines. Both compounds exerted similar effects, reducing the expression of multiple receptor tyrosine kinases and enhancing the effects of BRAF/MEK (Mitogen-Activated Protein Kinase Kinase) inhibitors [24]. In conclusion, melanoma cell lines appear to be more sensitive to BETi than some UCC or RCC. However, like in UCC, IC_50_ concentrations in melanoma cells were lower for JQ1 compared to PLX. Uveal melanoma cell lines were the most responsive studied entity yet, with IC_50_ doses around 0.25 µM PLX [50]. Cell cycle analysis of these cells revealed only small cell cycle alterations, but an increase of subG1 cells, similar as in our investigation of UCC. PARP cleavage was likewise described. The authors generated PLX-resistant sublines by long-term treatment which tolerated IC_50_ doses higher than 2 µM. Since none of the above studies investigated the impact on normal control cell lines, no comparison with our HBLAK control can be made. 

Surprisingly, no previous publication has reported on PLX effects regarding DNA damage induction or DNA damage repair, even though BRD4 is known to be involved in the regulation of DNA damage signaling and repair [51,52]. Multiple lines of evidence indicate that BRD4 is not only relevant for transcription regulation, but also as “a keeper of genome stability” [51]. Apart from functioning as a master regulator of many DNA repair components, BRD4 also exerts a non-transcriptional function in the control of DNA damage checkpoint activation and repair. BRD4 inhibition resulted in enhanced phosphorylated H2AX in prostate cancer cells because BRD4 is required for the repair of DNA DSBs induced by ionizing radiation [53]. Garcia et al. confirmed the induction of DNA damage by JQ1 in cholangiocarcinoma. Expression analysis after JQ1 treatment revealed reduced expression of cell-cycle regulators such as *MYC, TP53 (Tumor Protein P53), CHEK1, WEE1 (WEE1 G2 Checkpoint Kinase), CDK4, CDK6 (Cyclin Dependent Kinases),* and *E2F1 (E2F Transcription Factor 1),* and also of the HR component *BRCA2* [54]. Involvement of BRD4 in DNA damage checkpoint activation and DNA repair sets the stage for new cancer therapy approaches using combinations with DNA damaging compounds. Concurring with the above argument, we detected increased phosphorylation of H2AX in PLX-treated UCC, but less strongly in HBLAK cells. Likewise, the *GADD45* stress response markers were increased, particularly in sensitive VM-CUB1 cells, but not in normal HBLAK cells, suggesting that cancer cells may be more vulnerable than normal cells. Strong phosphorylation of H2AX in UCC but not in HBLAK may also further contribute to the observed differences in apoptosis response and caspase activation between cancerous and normal cells. Apart from its function in recruitment of DNA repair molecules γH2AX has also been reported to contribute to induction of apoptosis through the caspase-activated DNAse (CAD) pathway. Both γH2AX and caspase 3 activation are required for DNA fragmentation. γH2AX may regulate CAD activity that then results in DNA fragmentation [55]. 

Importantly, despite the evidence for DNA damage, we found checkpoint signaling to be inactivated in UCC, unlike in HBLAK where it was activated. Expression of the HR and NER DNA repair pathway as well as *PARP* genes were decreased by PLX. In contrast, NHEJ components were induced. Again, HBLAK cells responded less strongly or in the opposite direction. In conclusion, transcriptomic changes induced in UCC by PLX result in impeded DNA damage signaling, impaired repair of DNA double strand breaks by HR, and impaired repair of cisplatin induced DNA damage. Thus, we hypothesized that combined treatment with PLX and cisplatin may result in synergistic action. 

Since HR is impaired and *PARP* expression is reduced, while NHEJ is activated, PLX also established perfect conditions for epigenetically induced BRCAness in UC cells, but not in normal HBLAK cells. In conclusion, PLX treatment should sensitize UCC, but not normal cells, to PARPi. This may open up a therapeutic window for synergistic and tumor-specific treatment that may spare normal cells. Since there is a lack of data in the literature regarding epigenetically induced BRCAness in other normal cells, this issue should be further investigated in the future.

Patel et al. demonstrated by various approaches that active NHEJ repair is a major prerequisite for the cytotoxic action of PARPi in HR-deficient cells with a BRCAness phenotype [42]. Loss of PARP1 activity (and the PARP2 paralog with a similar function) may result in accumulation of DNA single-strand breaks that are converted to DNA double-strand breaks, which cannot be repaired in HR-deficient cells, leading to cell death. Accordingly, we additionally studied the combination of PLX with the PARP inhibitor talazoparib for synergistic action.

JQ1 was reported to induce DNA double strand breaks in pancreatic ductal adenocarcinoma, but at the same time to also decrease the expression of DNA repair proteins such as KU80 (Ku Autoantigen 80) and RAD51. The combination of JQ1 with the PARPi olaparib inhibited tumor growth in a patient-derived xenograft model more efficiently than the mono-treatments [56]. Ovarian carcinoma may become resistant to both cisplatin-based chemotherapy and PARPi through the restoration of the HR repair pathway. Combined BET inhibition resensitized these cancers to the treatment (reviewed in [57]). Mio et al. reported epigenetic induction of BRCAness in wild-type triple-negative breast cancer (TNBC) cells by BET inhibitors [27]. They confirmed a direct relation between BRD4 inhibition and reduced expression of the HR components BRCA1 and RAD51. BETi treatment increased cell death after treatment with cisplatin and resulted in synthetic lethality with PARPi. In our study, simultaneous combined treatment with PLX and cisplatin was highly synergistic in UCC. Even at significantly reduced concentrations, long-term proliferation was more strongly impaired than by the mono-treatments, and apoptosis was also more strongly induced. Since no synergism was detected in HBLAK control cells, these findings indicate a tumor-specific effect through the increased vulnerability of UCC. This tumor-specificity is explained by the observed differences in gene expression changes between UCC and HBLAK cells. 

A recent study by Zhang et al. investigated the combined treatment of pancreatic cancer cells with the dual HDAC/BET inhibitor TW9 and the chemotherapy compound gemcitabine, which is also a component of the cisplatin-based chemotherapy regimen for UC patients [58]. The authors described that sequential administration of gemcitabine and TW9 yielded additional synergistic anti-tumor effects. Thus, we investigated whether sequential treatment might also improve the anti-tumor effects of our combinations. However, in our study, sequential treatment impaired rather than improved synergistic action or affected normal HBLAK cells more strongly. While romidepsin also induced HR-deficiency in UCC, combined treatment of the HDACi with cisplatin acted also synergistically, but with less ideal combination indices and depending more strongly on the applied doses. Sequential treatment with PLX and gemcitabine was also not more efficacious. Taken together, we demonstrated that PLX highly efficiently episensitizes UCC to cisplatin treatment allowing for a significant reduction in toxicity in normal cells when the combination is simultaneously applied. 

Several PARP inhibitors, including talazoparib, were approved by the FDA as treatments for patients with deleterious germline *BRCA* mutations, e.g., ovarian, mammary or prostate cancers [59]. Talazoparib is similar to the best investigated PARP inhibitor, olaparib, but is around 100-fold more efficient in PARP trapping than olaparib [60]. Combined treatment of small-cell lung cancer cells with 0.5 µM talazoparib and the BETi I-BET762 resulted in synergistic effects on cancer cell viability [61]. In our study, talazoparib alone affected neither the viability of UCC nor HBLAK control cells strongly, with IC_50_ doses above 1 µM. However, PLX-resistant UM-UC-3 cells were strongly sensitized by PLX to the PARPi, resulting in impressive combination indices even at low doses. Long-term proliferation was almost abolished and apoptosis was more strongly induced in UM-UC-3 than by the combination of PLX and cisplatin, and even at reduced dosages that were tolerable by normal cells. Even sequential treatment with a reduced dosage remained significantly synergistic but was not beneficial compared to the simultaneous treatment. Taken together, combined treatment with the BETi PLX and the PARPi talazoparib appears to be a highly promising therapy approach for UC. 

## 5. Conclusions

In conclusion, we provide evidence that epidrugs like HDACi, and particularly the BET inhibitor PLX51107, can be applied to episensitize UC towards cisplatin-based chemotherapy, reducing toxicity to normal tissues. Furthermore, PLX induced changes that render UC cells highly sensitive to the PARP inhibitor talazoparib, allowing for the extension of PARPi efficacy to non-*BRCA1/2* mutated cancers.

## Data Availability

The data presented in this study is comprehensively shown in this article and its supplementary files. The underlying unprocessed raw data is available upon request.

## Supplementary Materials

The following are available online at https://www.mdpi.com/2072-6694/13/6/1376/s1, Figure S1: BRD4 expression across cell lines and treatment response towards BETi. Figure S2: Impact of PLX treatment on HBLAK cells. Figure S3: Long term impact of PLX treatment on cell cycle. Figure S4: Synergistic effects on UCC by romidepsin treatment combined with PLX or cisplatin. Figure S5: Effect of combinations treatment with PLX on long-term proliferation and synergy of sequential combination treatment. Table S1: Antibodies used in this study. Table S2: Primer and PCR conditions used in this study. Table S3: Cell line-specific IC_50_ doses of drugs applied in this study. Table S4: Fixed dose ratios applied for combination treatment.
