# Supplementary figures and images for: Epigenetic Treatment of Urothelial Carcinoma Cells Sensitizes to Cisplatin Chemotherapy and PARP Inhibitor Treatment
Source: Cancers (Basel). 2021 Mar 18;13(6):1376. doi: 10.3390/cancers13061376 (PMC8002916; doi:10.3390/cancers13061376)

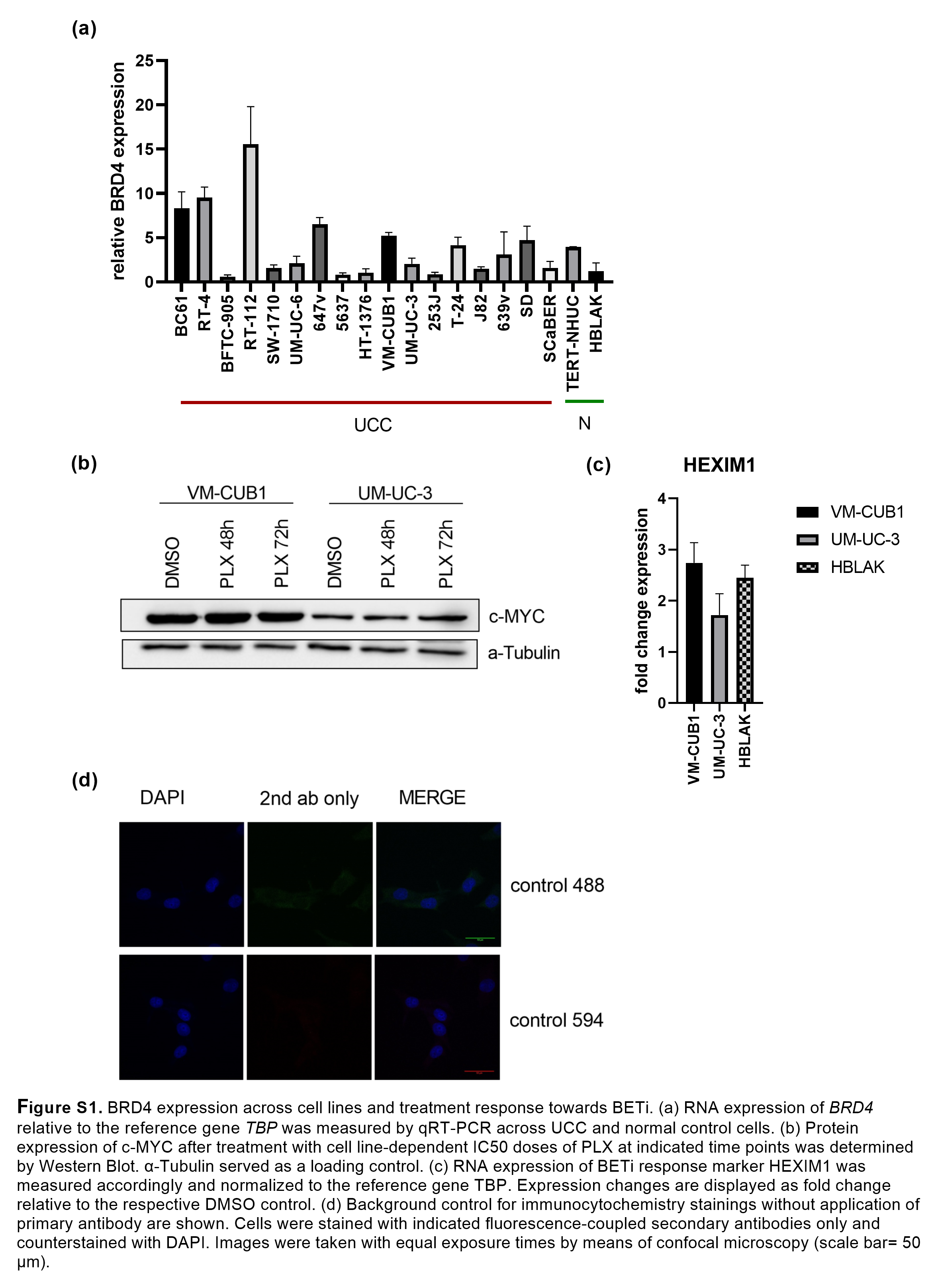

Supplement: Supplementary file 1 [file cancers-13-01376-s001.zip › supp files Thy/supp Figures/Figure S1.tif]

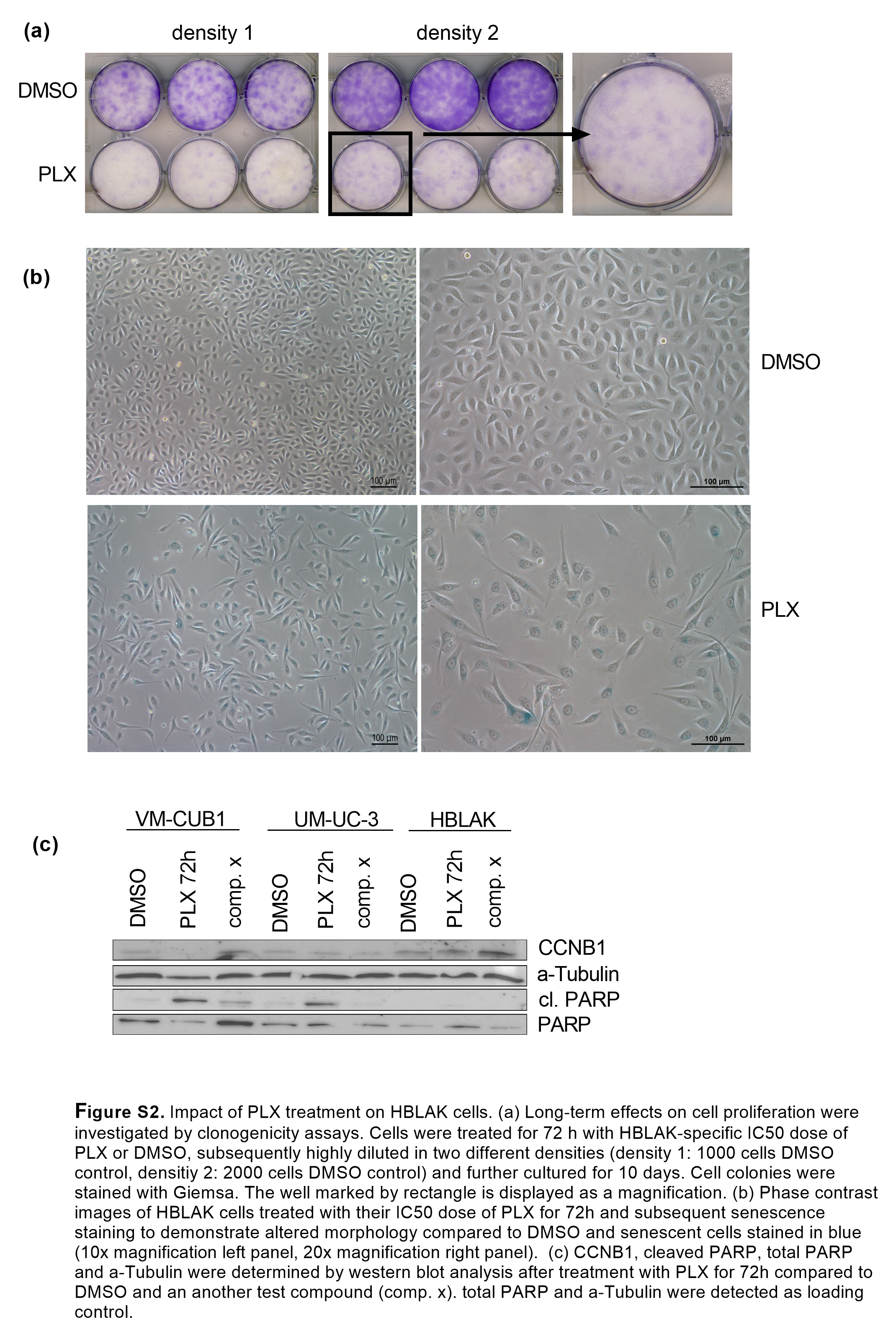

Supplement: Supplementary file 1 [file cancers-13-01376-s001.zip › supp files Thy/supp Figures/Figure S2.tif]

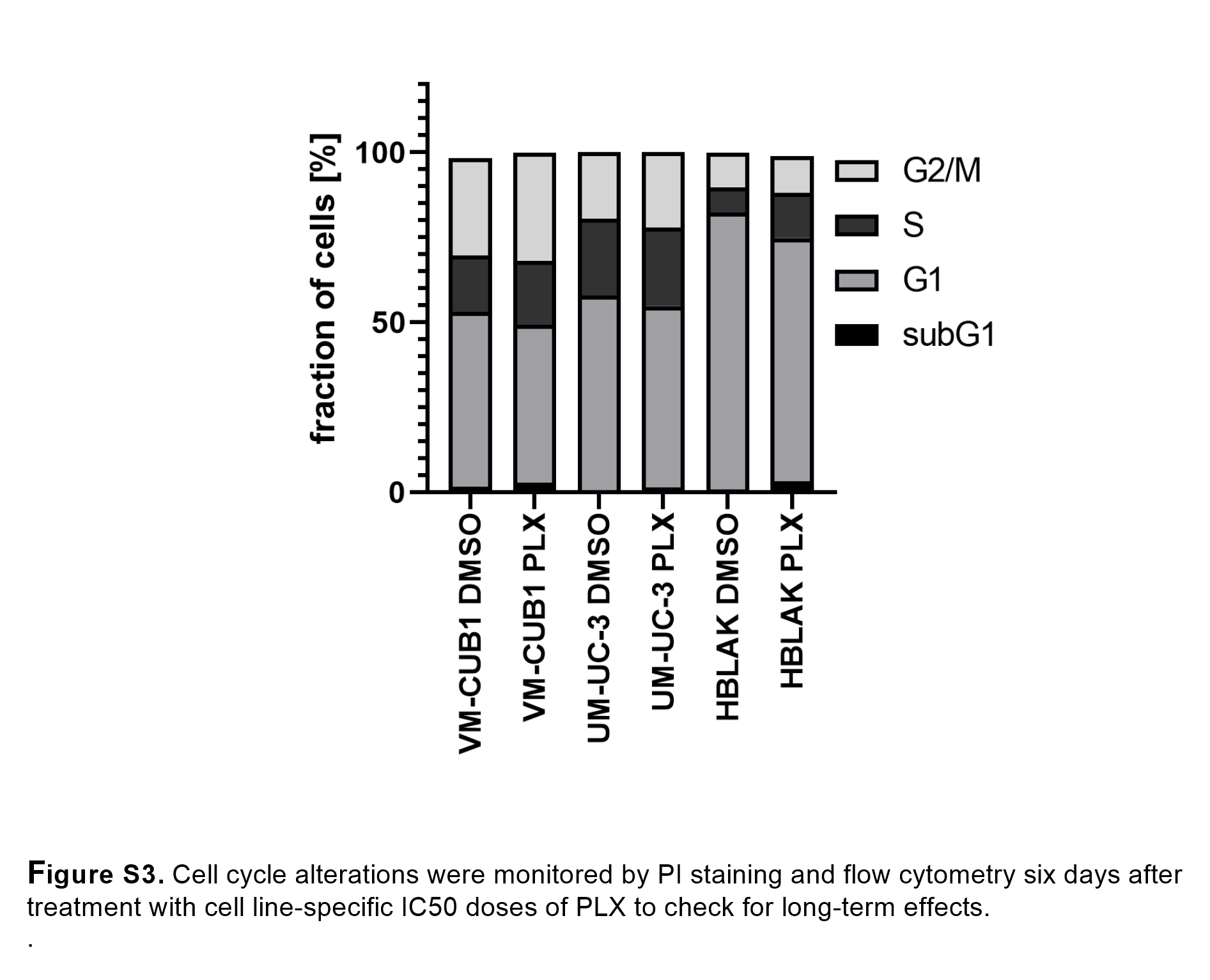

Supplement: Supplementary file 1 [file cancers-13-01376-s001.zip › supp files Thy/supp Figures/Figure S3.tif]

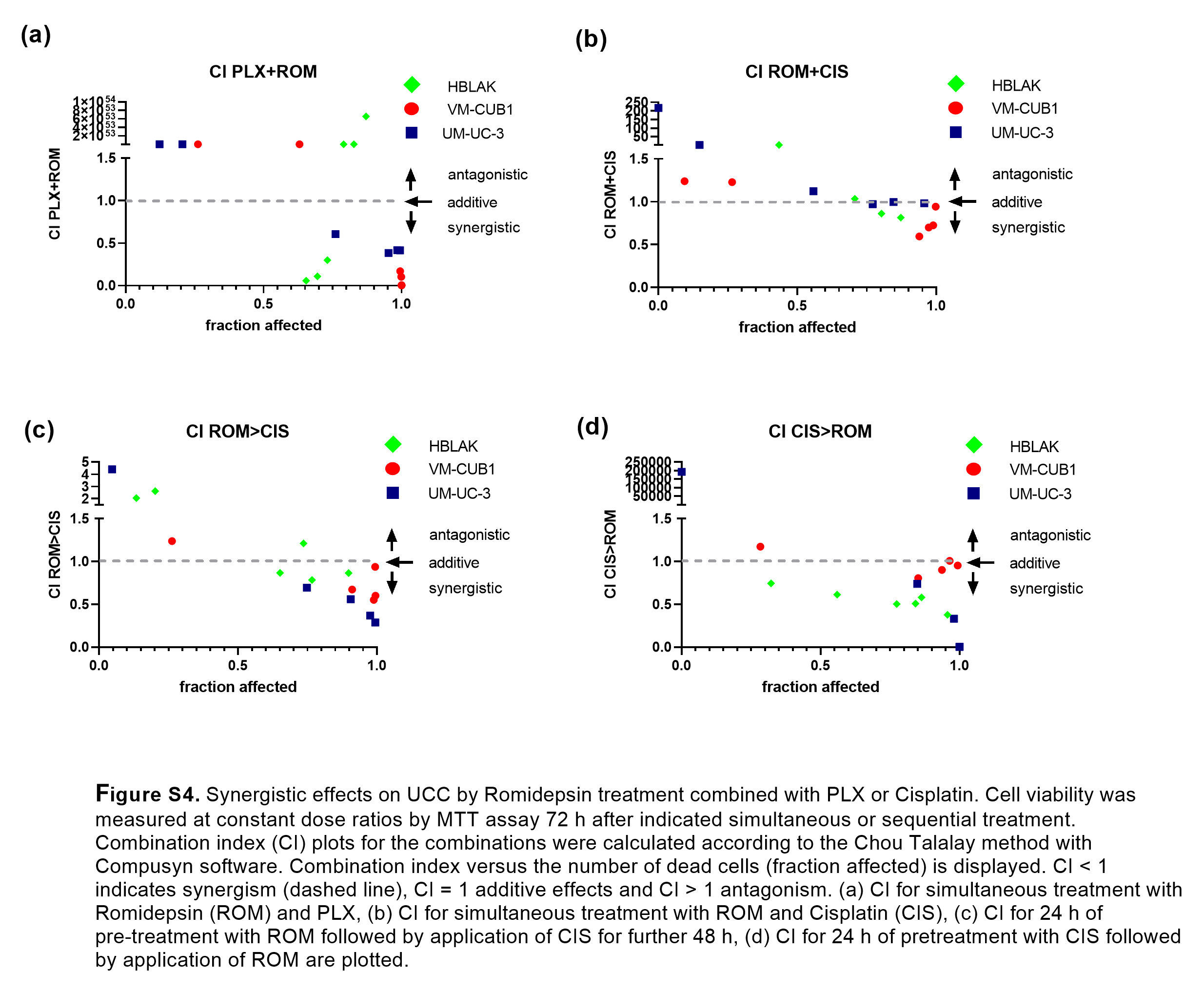

Supplement: Supplementary file 1 [file cancers-13-01376-s001.zip › supp files Thy/supp Figures/Figure S4.tif]

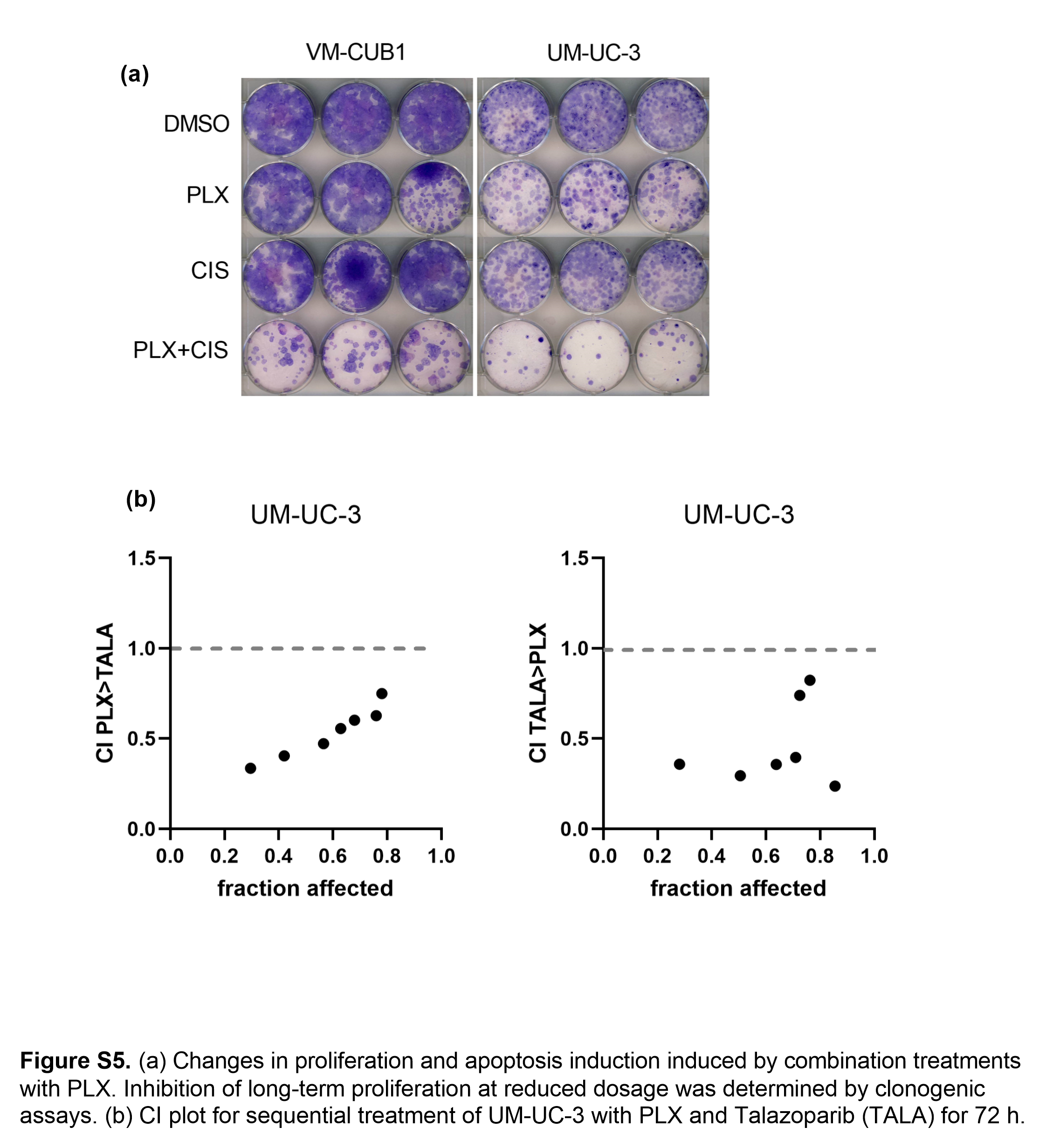

Supplement: Supplementary file 1 [file cancers-13-01376-s001.zip › supp files Thy/supp Figures/Figure S5.tif]
